# Naked mole‐rats are extremely resistant to post‐traumatic osteoarthritis

**DOI:** 10.1111/acel.13255

**Published:** 2020-10-28

**Authors:** Taketo Taguchi, Alexander Kotelsky, Masaki Takasugi, Martin Chang, Zhonghe Ke, Megan Betancourt, Mark R. Buckley, Michael Zuscik, Andrei Seluanov, Vera Gorbunova

**Affiliations:** ^1^ Departments of Biology University of Rochester Rochester NY USA; ^2^ Departments of Biology and Medicine University of Rochester Rochester NY USA; ^3^ Department of Biomedical Engineering University of Rochester Rochester NY USA; ^4^ Center for Musculoskeletal Research University of Rochester School of Medicine Rochester NY USA

**Keywords:** aging, naked mole rat, ostheoarthritis

## Abstract

Osteoarthritis (OA) is the most prevalent disabling disease, affecting quality of life and contributing to morbidity, particularly during aging. Current treatments for OA are limited to palliation: pain management and surgery for end‐stage disease. Innovative approaches and animal models are needed to develop curative treatments for OA. Here, we investigated the naked mole‐rat (NMR) as a potential model of OA resistance. NMR is a small rodent with the maximum lifespan of over 30 years, resistant to a wide range of age‐related diseases. NMR tissues accumulate large quantities of unique, very high molecular weight, hyaluronan (HA). HA is a major component of cartilage and synovial fluid. Importantly, both HA molecular weight and cartilage stiffness decline with age and progression of OA. As increased polymer length is known to result in stiffer material, we hypothesized that NMR high molecular weight HA contributes to stiffer cartilage. Our analysis of biomechanical properties of NMR cartilage revealed that it is significantly stiffer than mouse cartilage. Furthermore, NMR chondrocytes were highly resistant to traumatic damage. In vivo experiments using an injury‐induced model of OA revealed that NMRs were highly resistant to OA. While similarly treated mice developed severe cartilage degeneration, NMRs did not show any signs of OA. Our study shows that NMRs are remarkably resistant to OA, and this resistance is likely conferred by high molecular weight HA. This work suggests that NMR is a useful model to study OA resistance and NMR high molecular weight HA may hold therapeutic potential for OA treatment.

## Graphical abstract

Naked mole‐rats are remarkably resistant to post‐traumatic osteoarthritis. Their cartilage is stiffer than mouse cartilage and better protects chondrocytes from damage. These unique biomechanical properties of the naked mole‐rat cartilage are likely due to abundant high molecular weight hyaluronan present in the naked mole‐rat joints.

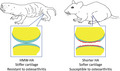

## 1 INTRODUCTION

Osteoarthritis (OA) is a chronic, degenerative joint disease characterized by severe pain associated with cartilage degeneration and joint inflammation (Martel‐Pelletier et al., 2016). Aging is a major risk factor for OA, and OA is a leading cause of disability in the elderly (Murray et al., 2012; Vos et al., 2012). OA is a degenerative disease with complex etiology, that results from age‐related cartilage degeneration and may also be associated with obesity (Mooney et al., 2011) and traumatic events such as sport‐related injuries (Englund, 2010; Roos et al., 1998). In the context of aging, the number of OA patients is predicted to increase as the proportion of elderly increases in the population. By year 2030, OA is predicted to affect one in four adults in the United States becoming the major cause of morbidity among individuals over 40 years of age (Hootman & Helmick, 2006). Current treatment options are limited to pain management, physical therapy, and end‐stage surgery. Thus, there is an unmet need to identify disease‐modifying treatments for OA.

Hyaluronic acid (HA) is a ubiquitous molecule that has been studied in joint biology and OA, with viscous solutions of HA clinically employed as intra‐articular injections to serve as a lubricant between the surfaces of articular cartilage and synovial joints. In vitro studies show that HA's lubrication properties increase as molecular weight of HA increases (Kwiecinski et al., 2011). In addition, molecular weight and concentration of HA in joint spaces are known to decrease in OA patients (Balazs, 1974; Balazs et al., 1967). Therefore, as mentioned, intra‐articular injections of high molecular weight HA have been used to alleviate OA symptoms (Hunter, 2015). Mice with the knockout of Hyaluronan synthase 2 (*Has2*) die in mid‐gestation (Camenisch et al., 2000). Conditional inactivation of Has2 results in skeletal deformities and a decrease in proteoglycan aggrecan deposition into the ECM (Matsumoto et al., 2009). Aggrecan degradation is the hallmark of cartilage degeneration in OA, and mice hypomorphic for aggrecan show increased incidence of spontaneous OA (Alberton et al., 2019).

The naked mole‐rat (NMR), *Heterocephalus glaber*, has received significant attention as an animal model of healthy aging (Ruby et al., 2018). NMR has the maximum lifespan of more than 30 years and shows resistance to multiple age‐related diseases (Buffenstein, 2005, 2008; Lagunas‐Rangel & Chavez‐Valencia, 2017; Lewis et al., 2016). NMRs are exceptionally resistant to cardiovascular diseases (Grimes et al., 2014) and cancer (Buffenstein, 2008; Delaney et al., 2013), two leading causes of death in humans.

Another unique feature of NMRs is that they accumulate high levels of very high molecular weight HA in their tissues (Tian et al., 2013). We have previously shown that high molecular weight HA is a key mediator of NMR's cancer resistance (Tian et al., 2013). Hence, in this study, we hypothesized that NMRs can be protected from OA due to their unique resistance to age‐related disease and the high levels of high molecular weight HA in the joints. HA is not only a filler of joint spaces but also is a major component of cartilage itself. However, it is not known whether HA molecular weight in cartilage could influence OA development. In cartilage, HA is assembled with proteoglycans, and the HA‐proteoglycan assemblies are further interwound with collagen fibers. We hypothesized that the mechanical properties of such biopolymer matrix may be affected by the molecular weight of HA. Indeed, in polymer science it is well‐known that a polymer material made of long polymer is stiffer than materials made of shorter polymer (Hendrikson et al., 2015; Jaspers et al., 2014). During OA progression, cartilage becomes softer (Kleemann et al., 2005); hence, stiffer cartilage of the naked mole‐rat composed of very high molecular weight HA may protect it from OA.

Here, we investigated vulnerability of resident cells and biomechanics of NMR cartilage, as well as NMR's resistance to OA using meniscal‐ligamentous injury (MLI) model. We found that in NMRs in situ chondrocytes are less vulnerable, the cartilage is intrinsically stiffer and remarkably resistant to OA as compared to mouse cartilage. Our results suggest that high molecular weight HA protects from OA and that NMR represents a unique model to study OA resistance.

## 2 MATERIALS AND METHODS

### 2.1 Animals

All animal experiments were approved and performed in accordance with guidelines set up by the University of Rochester Committee on Animal Resources. Naked mole‐rats were from the University of Rochester colonies. C57BL/6 mice were purchased from Charles River Labs. Tissues were obtained from at least three different animals for each experiment. All animals were young adults: NMRs were 2.5 years old, and mice were 6 months old. In the MLI study, all mice were males, and NMRs were two males and one female.

### 2.2 Quantification of molecular weight of HA in cartilage

In order to detect differences in molecular weight of HA in cartilage of C57BL/6 mice and NMRs, HAs were isolated from femoral and tibial cartilage and analyzed by pulse‐field gel electrophoresis following a previously established method (Tian et al., 2013).

### 2.3 Specimen preparations

To analyze cell vulnerability and Young's modulus of the extracellular matrix (ECM), distal femurs with fully intact cartilage were carefully dissected immediately after sacrifice using a previously established method (Kotelsky et al., 2019). Briefly, the intact cartilage on femoral condyles was exposed by carefully removing soft tissues (muscle, ligaments, and menisci) around the joint. Then, the bones were cut ~8 mm above the knee, and the dissected specimens were placed into Hank's Balanced Salt Solution (HBSS) with osmolarity adjusted to 303 ± 1 mOsm and a physiological pH of 7.4. The specimens were kept hydrated in HBSS throughout the cell vulnerability and ECM mechanical properties quantification experiments.

### 2.4 Quantification of young's modulus of articular cartilage

To investigate the possible effect of cartilage HA on mechanical properties, C57BL/6 mice (*n* = 5) and NMRs (*n* = 3) were used to quantify and compare the ECM Young's modulus (E) using a previously established inverse finite element‐based method (Kotelsky et al., 2018). Briefly, cartilage of the dissected specimens was fluorescently stained with 10 µg/ml of 5′‐DTAF (Sigma‐Aldrich) for 1 h at 23°C followed by a wash in iso‐osmotic HBSS (303 mOsm, pH = 7.4) to eliminate unbound fluorescent dye. The specimens were then placed on a custom microscope‐mounted testing device with the cartilage on the distal femoral condyles against the cover glass (Figure 2a). Cartilage on the medial femoral condyles was imaged under laser scanning confocal microscope (Olympus FV1000) with a 40× dry lens (LUCPLFLN, NA = 0.6) before and 5 min after a static load of 0.5 N was applied on top of the specimens, resulting in 3D confocal z‐stacks with a resolution of 0.31 µm/pixel in *x*‐*y* plane and 2.18 µm/slice along the z‐direction (Figure S1). Note that according to load distribution analysis (see “Assessment of contact forces on medial femoral condyles of C57BL/6 mice and NMRs”), the 0.5 N force induced 0.19 N and 0.22 N contact forces between the cover glass of the testing device and the cartilage on medial femoral condyles of C57BL/6 mice and NMRs, respectively (vide infra). The confocal z‐stacks were used to obtain tissue stretch maps (*λ*_z_) and measure the peak compressive deformation (i.e., minimal tissue stretch $\lambda_{z}^{\min}$). The tissue stretch maps were quantified by dividing the spatially varying thickness of the compressed cartilage (under a load of 0.5 N) by the thickness measured before compression (0 N). Stretch calculations were performed after fitting the normalized depth‐wise intensity profiles of the acquired confocal z‐stacks (before and during cartilage compression) with Gaussian curves using a previously established MATLAB algorithm (Kotelsky et al., 2018). The resulting spatially varying fit parameter *σ* (indicate depth‐wise intensity spread) is proportional to the cartilage thickness and was used to obtain stretch maps by dividing *σ* at each location after compression by the value of *σ* at the same location before loading (*λ*_z_ = *σ*_0.5 N_/*σ*_0 N_). Peak cartilage compressive deformation ($\lambda_{z}^{\min}$) was obtained from the *λ*_z_ maps by circumferentially averaging tissue stretch values observed within 5 µm from the global minima.

Specimen‐specific 3D finite element models (FEMs) of murine/NMR cartilage on medial condyles were constructed in FEBio (Maas et al., 2012) to determine the Young modulus of the cartilage ECM in tested specimens using a previously established method (Kotelsky et al., 2018). The cartilage on medial condyles was approximated as a uniform hemispherical shell with the cartilage thickness (42.8 ± 1.5 µm for C57BL/6 mice, 47.5 ± 3.9 µm for NMRs), and outer radius of curvature (913.8 ± 204 µm for C57BL/6 mice, 882.1 ± 5.7 µm for NMRs) measured from confocal z‐stacks acquired at baseline. The cartilage thickness was measured in ImageJ, and the radius of curvature was quantified in MATLAB by fitting a circle to the curvature of articular surface. The FEMs were constrained from movement in any directions on the top/inner surface (the bone‐cartilage interface), while the bottom/outer surface (the articular surface) was allowed to freely deform. The boundary displacement (*u*_z_), defined as the difference between the thicknesses of the compressed and resting‐state cartilage, was calculated from the experimentally measured peak tissue stretch ($u_{z}=\left(1‐\lambda_{z}^{\min}\right)\times \text{Thickness}\;\text{at}\;\text{the}\;\text{baseline}$). The boundary displacement was prescribed to a rigid platen at the cartilage‐glass interface along the z‐direction. The cartilage of both C57BL/6 mice and NMRs was modeled as a neo‐Hookean hyperelastic material with a Poisson ratio (*ν*) of 0.2 (Cao et al., 2006). The Young moduli of the articular cartilage on medial condyles were determined through an iterative parameter optimization algorithm implemented in FEBio by matching the experimentally measured forces (0.19 N and 0.22 N for C57BL/6 and NMRs, respectively) to the reaction forces determined from the corresponding FEMs on the rigid platen (Figure S2).

### 2.5 Assessment of contact forces on medial femoral condyles of C57BL/6 mice and NMRS

The application of an 0.5 N load on top of the dissected specimens resulted in 3 distinct contact points: two cartilage‐glass and one bone‐glass contact. The contact forces on the medial femoral condyles used to quantify Young's modulus were measured using a previously established method (Kotelsky et al., 2018). Briefly, the locations of three contact points (medial condyle‐glass, lateral condyle‐glass, and bone‐glass contacts) and the contact between the 0.5 N weight and the top of the specimen were first determined by placing the specimen on a sheet covered with black ink prior to compressing with the 0.5 N weight, which also was stained with black ink. The ink‐stained contact areas were then imaged using both inverted and upright microscopes. The contact locations, assessed from the acquired micrographs, were used to quantify reaction forces on medial femoral condyles through a moment balance equation. Mean values of the reaction forces on medial femoral condyles of C57BL/6 mice (0.19 N) and of NMRs (0.22 N) (Figure S3) were used to quantify and compare Young's moduli in these two groups.

### 2.6 Assessment of chondrocyte vulnerability to injurious loads

Differences in mechanically induced chondrocyte vulnerability between C57BL/6 mice and NMRs were assessed using a previously established method (Kotelsky et al., 2019). Briefly, the dissected specimens were vitally stained for 30 min at 37°C with 10 µM calcein AM (Life Technologies), an indicator of intact cell membranes (i.e., live cells). The specimens were then washed in HBSS for at least 10 min to wash out the excess calcein AM from the tissue. Next, the specimens were placed on the same mechanical testing device used to quantify the Young modulus of articular cartilage (Figure 2a) and statically loaded for 3 min with 0.5 N weight applied on top of the specimen (*n* = 7 for C57BL/6 mice, *n* = 3 for NMRs). The loading duration of 3 min was assumed to be sufficient for the ECM of both types of animals to reach poroelastic equilibrium based on the calculated gel diffusion time *t*_gel_, a measure of time constant for poroelastic equilibrium (Armstrong et al., 1984). In particular, *t*_gel_ was approximated as $\frac{\left(1+\nu \right)\left(1‐2\nu \right)h^{2}}{E\left(1‐\nu \right)k}$ (Armstrong et al., 1984) with equal to 42.8 µm and 47.5 µm (the average cartilage thickness measured in the current study), *E* equal to 6.95 MPa and 22.18 MPa (the average Young's moduli measured in the current study), permeability *k* = 1.1 × 10^−16 ^m^4^/N‐s and Poisson ratio *ν* = 0.2 based on previous studies (Cao et al., 2006), yielding values of 2.4 s and 0.9 s for articular cartilage in C57BL/6 mice and NMRs, respectively. The articular surfaces of femoral condyles were imaged under appropriate fluorescent illumination with a 4× dry lens (NA = 0.13) on an Olympus IX‐81 before the application of 0.5 N load and 5 min after the load was removed. The specimens were then stained with 40 µg/ml propidium iodide (PI, Life Technologies), an indicator of permeabilized cell membranes, and re‐imaged. The resulting fluorescence micrographs acquired after compression were compared with the micrographs obtained at baseline. The areas within which cells lost calcein AM fluorescence and became PI‐positive (i.e., the areas of cell injury/death) were quantified in ImageJ (Schneider et al., 2012).

### 2.7 Assessment of cartilage degeneration after meniscal‐ligamentous injury

Post‐traumatic OA was surgically induced on a limb by employing a meniscal‐ligamentous injury (MLI) model (Hamada et al., 2014). Briefly, this model dissects small part of anterior horn of meniscus, and that leads to the development of post‐traumatic OA joint changes by 4 weeks post‐injury, with progression to terminal disease by 4 months in mice. In our study, animals were administered MLI injury, and at 12 weeks post‐MLI, joints were harvested. In both mice and NMRs, the contralateral limb provided a sham control. Tissue fixation and histology preparation were performed by a previously established systematic approach (Sampson et al., 2011).

### 2.8 Statistical analysis

The extent of cartilage deformation, cartilage ECM Young's moduli, areas of injured/dead cells, and percent loss of uncalcified cartilage layer after MLI was compared between C57BL/6 mice and NMRs using a Student's *t* test conducted in GraphPad Prism (version 6.01).

## 3 RESULTS

### 3.1 NMR cartilage is composed of high molecular weight HA and is stiffer than mouse cartilage

**FIGURE 1:**
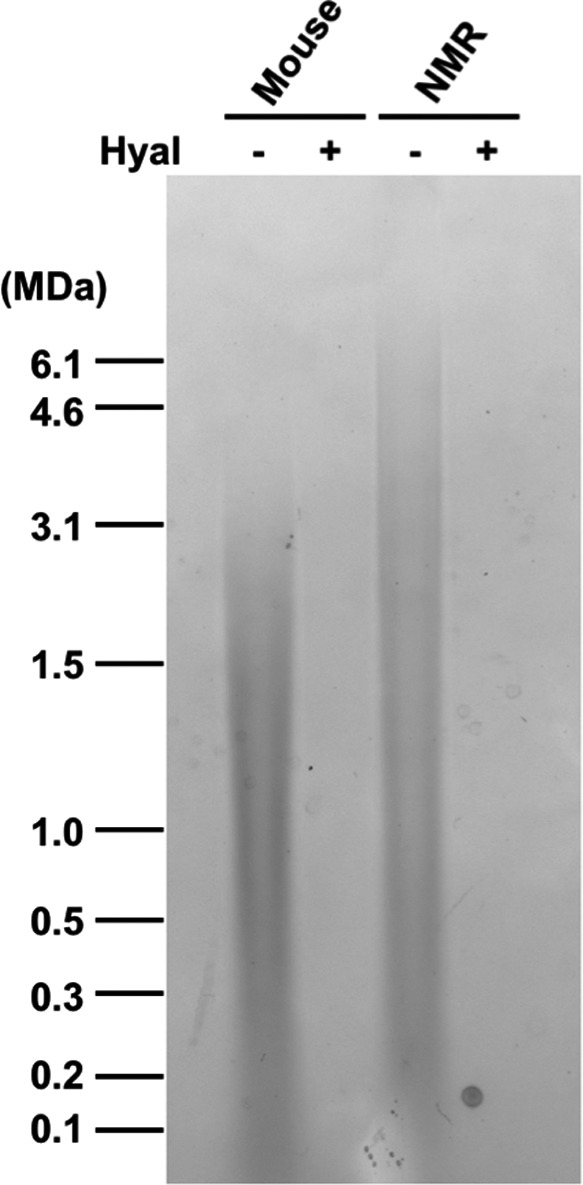
Naked mole‐rat cartilage is composed of higher molecular weight HA than mouse cartilage. Purified HA separated on pulse‐field gel. Samples were either run intact or pre‐digested with hyaluronidase (Hyal).

We previously reported that HA in NMR tissues (skin, heart, kidney, and brain) has higher molecular weight than in mice (Tian et al., 2013). However, NMR cartilage had not been previously analyzed. Using pulse‐field electrophoresis, we first examined the molecular weight of HA extracted from mouse and NMR cartilage. We found that HA in NMR cartilage had a molecular weight of, at least, twofold higher than the murine form (Figure 1).

**FIGURE 2:**
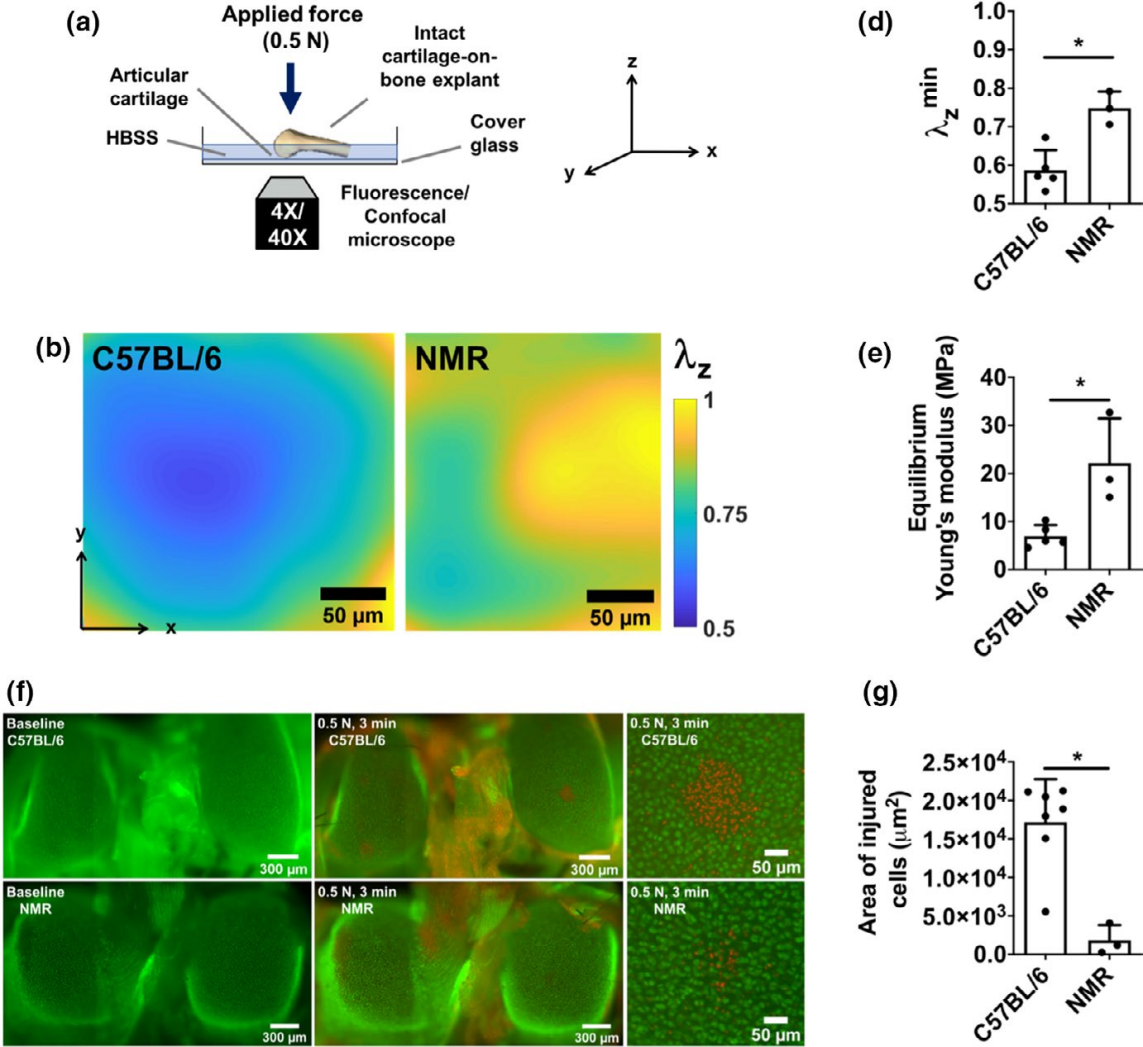
Naked mole‐rat (NMR) cartilage is stiffer than mouse counterpart, and NMR chondrocytes are better protected from trauma‐induced cell deaths. (a) Schematic representation of the experimental setup for and strain mapping and trauma‐induced chondrocyte deaths experiments. (b, c) Stretch mapping experiment: (b) Stretch maps (*λ*_Z_) of condyles after compression where the degrees of compression were represented by colors: *λ*_Z_ = final thickness/initial thickness of the stained cartilage. Lower stretch means more compression; (c) Quantification of cartilage deformation; (d) Solid matrix Young's modulus of mouse and NMR calculated using a finite element model. (e, f) Trauma‐induced chondrocyte deaths experiment: (e) Representative fluorescent microscopy images of mouse and NMR cartilages (live cells: green; dead cells: red). Left: before traumatic loading; Middle: after traumatic loading; Right: the zoomed images of the area of trauma. (f) Analysis of chondrocyte death area. Data are mean + SD; *Statistical significance *p* < 0.05.

We next compared mechanical properties (Young's modulus) of mouse and NMR cartilage using a previously developed method (Kotelsky et al., 2018) involving comparison of confocal micrographs of stained condyles before and after application of load (Figure 2a). According to representative stretch maps (Figure 2b) and analysis of the full data set (Figure 2c), NMR cartilage exhibited significantly less tissue deformation (i.e., higher $\lambda_{z}^{\min}$) under an applied static load of 0.5 N. Consequently, the Young modulus (i.e., intrinsic stiffness) of NMR cartilage was significantly higher than in C57/BL6 mice (*E* = 22.18 MPa vs. *E* = 6.95 MPa) (Figure 2d).

### 3.2 NMR chondrocytes are resistant to traumatic damage

We tested whether NMR chondrocytes are more protected from traumatic injuries using the setup shown in Figure 2a. When a static load of 0.5 N was applied on top of the dissected femurs for 3 min, substantially smaller areas of dead cells (~10‐fold smaller) were observed on the surface of NMR condyles compared to C57BL/6 mouse specimens (Figure 2e,f). Taken together, these results demonstrate that the ECM in NMR cartilage is stiffer and NMR chondrocytes are more protected from damage than in C57BL/6 mice. These findings suggest that NMR may be protected from OA.

### 3.3 NMR is resistant to post‐traumatic OA

**FIGURE 3:**
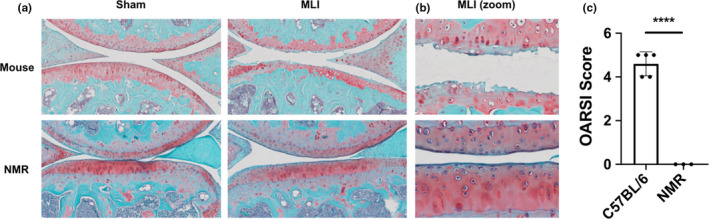
Naked mole‐rats (NMR) are resistant to post‐traumatic OA. (a) Representative images of sham and MLI joints sagittal sections of mouse and NMR, 12 weeks post‐injury, stained with Safranin O/Fast Green. (b) Larger magnification of the MLI images shown in (a). (c) OARSI scoring of the sections. *n* = 5 animals for mouse; *n* = 3 animals for NMR. Data are mean + SD; ****Statistical significance *p* < 0.0001.

To test whether NMR is resistant to OA, we employed MLI model of post‐traumatic OA, in both NMR and mouse. By surgical transection of the medial collateral ligament and removal of a part of the anterior horn of the medial meniscus, the knee joint becomes destabilized and post‐traumatic OA ensues. The development of OA, evidenced by articular cartilage degeneration, is depicted by representative histologic images and staged via OARSI scoring. As shown in Figure 3, 12 weeks after MLI, mouse cartilage exhibited degenerative changes as typically seen in the MLI model in 12 weeks post‐injury: loss of uncalcified layer; depletion of proteoglycan staining; fibrillation and thinning of cartilage. Remarkably, NMR cartilage showed no signs of degenerative changes in the MLI group. This result shows that NMR is extremely resistant to OA, which is the one of most prevalent age‐related diseases.

## 4 DISCUSSION

In the present study, we investigated the biomechanics of NMR cartilage and the resistance of NMRs to the development of OA. We demonstrated that NMR cartilage is composed of higher molecular weight HA; NMR cartilage is intrinsically stiffer; NMR chondrocytes are less vulnerable to traumatic injury; and NMRs are more resistant to OA compared to mice. These results suggest that high molecular weight HA in cartilage may be a key factor in preventing cartilage degradation in OA.

There are two main reasons NMR cells secrete higher molecular weight HA than mouse and human cells (Tian et al., 2013). NMRs have two unique amino acid changes in the hyaluronan synthase *HAS2* gene and show the increased HAS2 protein levels compared with human and mouse cells. In addition, NMR cells have low hyaluronidase activity. Because of those combined effects of the increased HA anabolism and decreased HA catabolism, the molecular weight of HA in NMR tissues is higher than in any other mammal examined (Tian et al., 2013). In the present study, we confirmed that NMR cartilage is also composed of much higher molecular weight HA compared to mouse cartilage (Figure 1).

Using the recently developed method to study biomechanics of whole‐mount distal femoral cartilage, we demonstrated that NMR cartilage is greater than threefold intrinsically stiffer than the cartilage of C57BL/6 mice (Figure 2). This difference in cartilage material stiffness led to smaller cartilage deformation in NMRs under 0.5 N static loading. Since the extent of tissue deformation scales with the extent of cell injury/death (Chen et al., 2003; Levin et al., 2005), intrinsically stiffer cartilage of NMRs significantly reduced vulnerability of chondrocytes to traumatic cell death, exhibiting chondroprotective effects against mechanical injury as compared to mice (Figure 2e,f). Importantly, health of articular chondrocytes is crucial for maintaining cartilage homeostasis and thus may play a role in preventing injury‐induced OA. In addition, stiffness of human cartilage was reported to decrease with the progression of OA (Kleemann et al., 2005), suggesting that stiff NMR cartilage may be protective from OA.

By employing an MLI injury model, we demonstrated that NMRs are extremely resistant to post‐traumatic OA. NMRs presented no signs of cartilage degradation, while mice subjected to the same injury presented severe OA phenotypes including significant cartilage loss.

Importantly, NMRs are highly active animals and the lack of cartilage degradation cannot be explained by the lack of movement. In our facility, NMRs move around in the habitats composed of multiple chambers connected by tubes. Following MLI injury, the experimental animals were returned to their native colonies and continued their active lifestyle. Since mice were housed in standard cages, their movements were more limited than NMRs, which would give them an advantage in recovering from the injury. Therefore, the observed resistance of NMR to OA is best explained by the decreased cell vulnerability and by the increased mechanical strength of NMR cartilage due to the presence of high molecular weight HA. Another contributing factor is likely to be NMR high molecular weight HA in the synovial fluid, as lubrication properties of HA increase as molecular weight of HA increases (Kwiecinski et al., 2011), and thereby further reducing vulnerability of in situ chondrocytes.

Cellular senescence has been implicated in the pathogenesis of osteoarthritis (Deng et al., 2019; Jeon et al., 2017) by promoting inflammation. Although NMRs cells undergo senescence in response to stress this response is attenuated (Zhao et al., 2018). Reduced senescence in NMR joints may be another factor protecting from OA. As HMW‐HA has cytoprotective properties (Takasugi et al., 2020), it may be acting to attenuate senescence. Finally, there may be systemic factors contributing to NMR OA resistance, such as lower levels of inflammatory response, as high molecular weight HA downregulates inflammation (Petrey & de la Motte, 2014).

To unequivocally prove that NMR high molecular weight HA is responsible for OA resistance, it would be important to generate a transgenic mouse model expressing NMR‐size HA in cartilage and test whether it confers mechanical advantages and post‐traumatic OA resistance. Overexpression of NMR version of HAS2 in chondrocytes would suffice to test this. However, since mouse tissues rapidly degrade HA, achieving the same molecular weight and abundance of HA in the mouse as observed in the NMR, would also require inhibiting hyaluronidase genetically or pharmacologically.

Our study suggests that increasing HMW‐HA in the joints may have therapeutic effect. Although intra‐articular injections of HA have been used in the clinic, more efficient and less invasive methods may be desirable. These may include gene therapy vectors expressing HAS2 enzyme delivered directly to the joint, or systemic inhibitors of hyaluronidases that would slow down the degradation of HMW‐HA leading to accumulation of HMW‐HA in both cartilage and synovial fluid.

In summary, our study demonstrates that NMRs are resistant to yet another age‐related disease, OA. Our results show that NMR cartilage is composed of very high molecular weight HA that is significantly stiffer than the mouse cartilage, which is composed of lower molecular weight HA. These results suggest that very high molecular weight HA in cartilage protects NMRs from OA. Intra‐articular injections of HA have been used to treat OA in human patients; however, the length of HA used in these treatments is typically 1 MDa, which is shorter than HA in NMR cartilage. Our work may encourage further investigation into the beneficial effects of very high molecular weight HA both in cartilage and synovial fluid for maintaining cartilage biomechanics and potential therapeutic interventions to delay or prevent OA progression.

## CONFLICT OF INTEREST

Authors declare no conflict of interest.

## AUTHOR CONTRIBUTIONS

T.T. and A.K. designed research and performed most of the experiments; M.T. analyzed hyaluronan; M.C. performed animal surgery; Z.K. and M.B. performed experiments; M.R.B. and M.Z. designed research; A.S and V.G. designed and supervised research; T.T and V.G. wrote the manuscript with input from all authors.

## Data Availability

The data that support the findings of this study are available in the main text and supplementary material (Figures S1–S3) of this article.
