# Supplementary material for: Naked mole‐rats are extremely resistant to post‐traumatic osteoarthritis
Source: Aging Cell. 2020 Oct 28;19(11):e13255. doi: 10.1111/acel.13255 (PMC7681040; doi:10.1111/acel.13255)
Supplement: Supplementary file 1 [file ACEL-19-e13255-s001.pdf]

Supplementary Figures

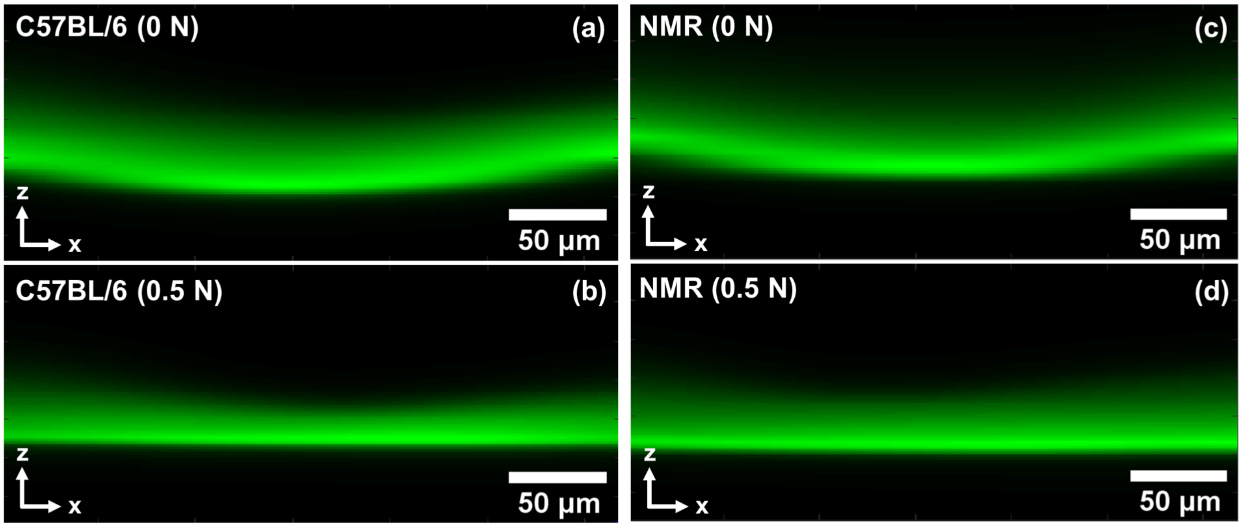

Figure S1. Representative sagittal cross sections obtained from confocal z-stacks of C57BL/6 and NMR cartilages prior to compression (top row) and after application of 0.5 N (bottom row).

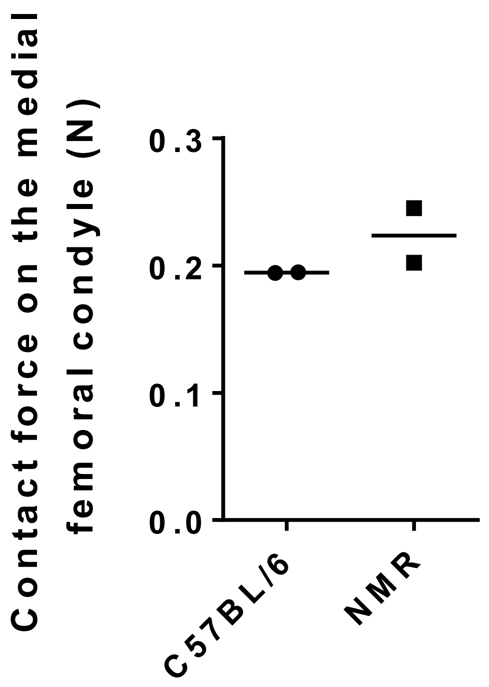

Figure S2. Measured cartilage-glass contact force on medial femoral condyles of C57BL/6 mice and NMRs (n = 2 specimens per group) induced by an 0.5 N load applied on top of the specimens. Each data point represents the contact force on medial femoral condyles of different specimens. The horizontal line indicates the mean contact force that was used for quantification of Young’s modulus.

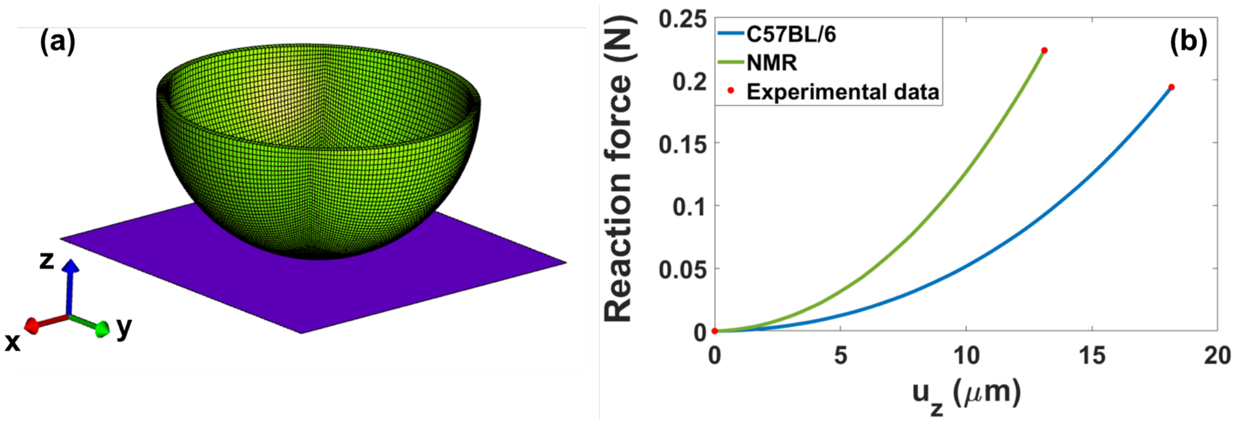

Figure S3. (a) Representative finite element model of cartilage on the medial femoral condyle. The tissue was modeled as an ideal hemispherical shell governed by a neo-Hookean, hyperelastic constitutive relationship. A rigid wall (flat plate) contact was applied with a prescribed displacement to compress the bottom (outer) surface in the positive z-direction. The top surface represents the cartilage–bone interface, which was constrained from any rotation and translation. (b) FEM-predicted behavior of reaction force versus displacement for C57BL/6 and NMR samples.
